# Evaluating the effects of sevelamer carbonate on cardiovascular structure and function in chronic renal impairment in Birmingham: the CRIB-PHOS randomised controlled trial

**DOI:** 10.1186/1745-6215-12-30

**Published:** 2011-02-02

**Authors:** Colin D Chue, Jonathan N Townend, Richard P Steeds, Charles J Ferro

**Affiliations:** 1 Department of Cardiology, Queen Elizabeth Hospital and University of Birmingham, Birmingham, UK; 2 Department of Nephrology, Queen Elizabeth Hospital and University of Birmingham, Birmingham, UK

## Abstract

**Background:**

Serum phosphate is an independent predictor of cardiovascular morbidity and mortality in patients with chronic kidney disease and the general population. There is accumulating evidence that phosphate promotes arterial stiffening through structural vascular alterations such as medial calcification, which are already apparent in the early stages of chronic kidney disease.

**Aim:**

To determine the effects of phosphate binding with sevelamer carbonate on left ventricular mass and function together with arterial stiffness in patients with stage 3 chronic kidney disease.

**Methods/Design:**

A single-centre, prospective, randomised, double-blind, placebo-controlled trial of 120 subjects with stage 3 chronic kidney disease recruited from University Hospitals Birmingham NHS Foundation Trust. Baseline investigations include transthoracic echocardiography and cardiac magnetic resonance imaging to assess ventricular mass, volumes and function, applanation tonometry to determine pulse wave velocity and pulse wave analysis as surrogate measures of arterial stiffness and dual energy x-ray absorptiometry scanning to determine bone density. During an open-label run in phase, subjects will receive 1600 mg sevelamer carbonate with meals for four weeks. They will then be randomised to either continue sevelamer carbonate or receive an identical placebo (60 subjects per arm) for the remaining 36 weeks. Four-weekly monitoring of serum electrolytes and bone biochemistry will be performed. All baseline investigations will be repeated at the end of the treatment period. The primary endpoint of the study is a reduction in left ventricular mass after 40 weeks of treatment. Secondary endpoints are: i) change in aortic compliance; ii) change in arterial stiffness; iii) change in arterial elastance; iv) change in left ventricular systolic and diastolic elastance; v) change in left ventricular function; and vi) change in bone density.

**Trial Registration:**

This trial is registered at ClinicalTrials.gov: NCT00806481 and Current Controlled Trials: ISRCTN35254279.

## Background

The risk of cardiovascular disease is elevated in patients with chronic kidney disease (CKD) with an inverse graded relationship to glomerular filtration rate (GFR) independent of other risk factors [1]. The magnitude of this excess risk varies according to age, but for patients with moderately impaired renal function at stage 3b CKD (GFR 30-44 ml/min/1.73 m^2^), cardiovascular risk is at least doubled [2]. Although cardiovascular risk in end stage kidney disease (ESKD) is elevated, the global burden of cardiovascular disease caused by early CKD is much greater in public health terms as approximately 10% of the general population have a GFR within the CKD stage 3 range (30-59 ml/min/1.73 m^2^) [3]. These individuals are much more likely to die from cardiovascular disease than progress to ESKD [4].

Atherosclerotic diseases such as myocardial infarction only account for a minority of cardiovascular deaths in patients with CKD, the remainder being attributable to congestive heart failure, sudden cardiac death and arrhythmia [5]. These appear to be driven by underlying structural abnormalities such as left ventricular hypertrophy, fibrosis and dysfunction, which are near universal in patients with CKD and are present even in the early stages of disease [6]. Recent work suggests that increased arterial stiffness plays a major role in the development of these myocardial abnormalities, and both increased left ventricular mass and increased arterial stiffness are of proven prognostic significance in CKD [7-10].

Serum phosphate is an independent predictor of cardiovascular morbidity and mortality in patients with CKD[11] and within the general population [12]. Reasons for this are unclear, but phosphate is intimately involved in the regulation of medial vascular smooth muscle growth and calcification giving rise to the possibility that it is acting as a mediator of increased arterial stiffness. Phosphate binders administered to control hyperphosphataemia in CKD might therefore be expected to reduce or slow the progression of arterial stiffness in addition to their primary role of preventing metabolic bone disease. The non-calcium-based phosphate binder sevelamer, which reduces hyperphosphataemia without increasing calcium-phosphate product, has near ideal pharmacological properties for such an action. Indeed, in three randomised controlled trials it has been shown to be superior to calcium-based phosphate binders in attenuating the progression of coronary artery and aortic calcification [13-15].

Although serum phosphate rises early in the course of CKD, phosphate binders are currently only used in late-stage disease when major abnormalities of calcium and phosphate metabolism are present. By this time it is likely that arterial and ventricular function are already significantly impaired, partly through prolonged exposure of the vascular system to high levels of phosphate. This study aims to examine the effect of phosphate binding with sevelamer on left ventricular mass, markers of arterial stiffness and left ventricular function in early stage CKD.

## Methods

### Hypothesis

Phosphate binding with sevelamer carbonate will reduce left ventricular mass, improve indices of left ventricular systolic and diastolic function, and reduce arterial and cardiac stiffness in patients with stage 3 CKD.

### Study Design

This is a single-centre prospective, randomised, double-blind, placebo-controlled trial of 120 subjects with stage 3 CKD (defined as an estimated GFR 30-59 ml/min/1.73 m^2^) established on conventional treatment with an angiotensin converting enzyme inhibitor or angiotensin receptor blocker for at least 3 months before enrolment. GFR will be estimated by the 4-variable Modification of Diet in Renal Disease formula with serum creatinine recalibrated to be traceable to an isotope derived mass spectroscopy method [16]. Inclusion and exclusion criteria are detailed in Table 1.

**Table 1:** Inclusion and exclusion criteria

Inclusion Criteria
Age 18-80 years
Chronic kidney disease stage 3 (estimated GFR 30-59 ml/min/1.73 m^2^)
Office BP controlled to <140/90 mmHg for ≥12 months before entry
Total cholesterol <5.5 mmol/L

**Exclusion Criteria**

Existing or previous treatment within the past year with a phosphate binder or vitamin D analogue
Hyperphosphataemia (serum phosphate >1.8 mmol/L)
Hypophosphataemia (serum phosphate <0.8 mmol/L)
Uncontrolled secondary hyperparathyroidism (PTH >80 pg/ml)
Diabetes mellitus
Pregnancy
Women of child-bearing age not on contraception
Bowel obstruction
Dysphagia or other swallowing disorder
Severe gastrointestinal motility disorders including severe constipation
Previous major gastrointestinal tract surgery

### Baseline Studies

**Figure 1:**
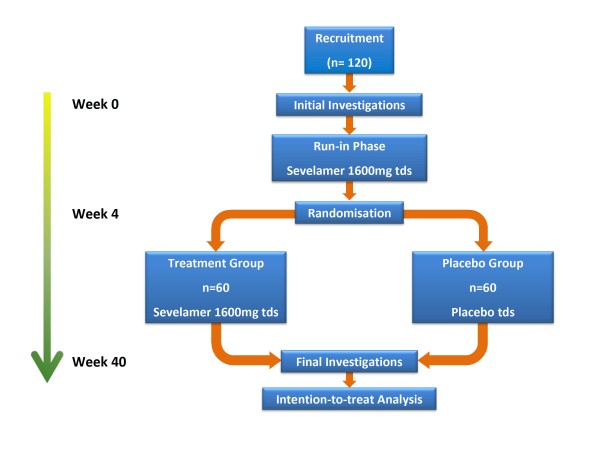
**Study timeline**. 120 subjects will undergo baseline investigations before entering a 4 week open-label run-in phase in which all subjects will receive 1600 mg of sevelamer carbonate with meals. Participants will then be randomised to continue treatment with sevelamer carbonate 1600 mg with meals or identical placebo for the remaining 36 weeks. Safety monitoring will be performed every four weeks. Investigations will be repeated at week 40 after which subjects will have completed participation in the study.

All subjects will undergo a baseline visit (Figure 1) during which the following will be performed: i) questionnaire regarding past medical history, drug history and tobacco and alcohol consumption; ii) clinical examination of all systems; iii) measurement of height, weight, hips and waist; iv) 12-lead electrocardiogram (ECG); v) office brachial blood pressure and heart rate measurement in triplicate using the non-dominant arm following 15 minutes of rest with a validated oscillometric sphygmomanometer (HEM-705CP, Omron, Henfield, United Kingdom[17] or Dinamap Procare 200, GE Healthcare, United Kingdom[18]) according to British Hypertension Society guidelines[19]; vi) applanation tonometry to determine carotid-femoral aortic pulse wave velocity; vii) applanation tonometry to derive central pressure waveforms from pulse wave analysis and to determine central systolic and diastolic pressures and augmentation index; viii) dual energy x-ray absorptiometry (DEXA) scanning to assess bone mineral density as recommended by the World Health Organisation[20,21]; ix) collection of serum and plasma following 30 minutes of supine rest for haematological and biochemical analysis including serum calcium, phosphate, 1,25-dihydroxyvitamin D and parathyroid hormone (PTH); x) storage of serum and plasma at -80°C for future assay of biomarkers associated with cardiovascular function and calcification; xi) collection of a spot urine sample to determine albumin: creatinine ratio; xii) storage of a spot urine sample at -80°C for future proteomic studies; xiii) transthoracic echocardiography to determine systolic and diastolic ventricular function and measures of ventricular-vascular interaction; xiv) a lateral plain abdominal radiograph to semi-quantitatively assess abdominal aortic calcification as previously described[22]; xv) cardiac magnetic resonance imaging (CMR) to determine ventricular volumes, function and mass and aortic distensibility as previously described[23]; xvi) 24-hour urine collection for determination of phosphate excretion; and xvii) 24-hour ambulatory blood pressure and heart rate monitoring with a validated ambulatory blood pressure monitor (ABPM-04, Meditech, Budapest, Hungary) [24]. Eligible female subjects of childbearing age will undergo urine pregnancy testing prior to entry into the study.

### Cardiac Magnetic Resonance Imaging

Cardiac magnetic resonance imaging will be performed using a 1.5-T scanner (Sonata Symphony, Siemens, Erlangen, Germany) with subjects in the supine position. Serial contiguous short axis cines will be piloted from the vertical long axis and horizontal long axis images of the left and right ventricles (ECG R wave-gated, steady-state free precession imaging [True-FISP]; temporal resolution 40-50 ms, repetition time 3.2 ms, echo time 1.6 ms, flip angle 60°, slice thickness 7 mm with 3 mm gap) in accordance with previously validated methodologies [23]. Analysis will be performed offline (Argus Software, Siemens, Erlangen, Germany) by a single blinded observer for the assessment of ventricular volumes (end-diastolic, end-systolic and stroke volumes), function (ejection fraction) and left ventricular mass [23,25]. Aortic distensibility will be assessed at the ascending aorta and proximal descending aorta at the level of the pulmonary artery and the distal descending aorta at the level of the diaphragm and calculated using previously validated formulae [26]. Brachial blood pressures will be simultaneously recorded at the time of scanning.

Dynamic tissue-tagging magnetic resonance imaging allows direct non-invasive assessment of regional systolic myocardial shortening and is a previously validated technique [27]. Spatial modulation of magnetization will be used to generate a uniform grid pattern with 8 mm tag separation on the left ventricular myocardium at three short axis sections (basal, equatorial and apex) and the horizontal long axis image using a fast field echo multi-shot sequence (temporal resolution 40-50 ms, repetition time 3.9 ms, echo time 4.4 ms, voxel size 1.8/1.3/6.0 mm^3^, flip angle 14°, tag grid angle 45° with slice thickness 6 mm and a minimum number of 15 phases per cardiac cycle) with prospective ECG gating as previously described [28]. The myocardial grid will be followed through systole for deformation caused by ventricular contraction. Blinded analysis will be performed offline for left ventricular longitudinal shortening and apical and basal rotation as previously described [28].

### Pulse Wave Velocity and Pulse Wave Analysis

Pulse wave velocity (SphygmoCor, AtCor Medical, Sydney) will be performed as previously described[29] following 15 minutes of supine rest using a high-fidelity micromanometer (SPC-301, Millar Instruments, Texas) to sequentially record ECG-gated carotid and femoral arterial waveforms. The path length will be calculated by subtracting the distance between the sternal notch and carotid recording site from the distance between the sternal notch and femoral site. This method is reproducible in both healthy subjects and patients with renal impairment [30,31]. Pulse wave analysis will be performed using the micromanometer to flatten, but not occlude, the radial artery using gentle pressure. Data will be collected directly into a portable computer and after 11 seconds of data capture, an averaged peripheral waveform and corresponding central waveform will be generated using a validated transfer function. The central waveform will be analysed using the system software to determine the augmentation index (AIx) and central aortic pulse and systolic pressures. AIx represents the difference between the second and first peaks of the central pressure waveform in systole, expressed as a percentage of the pulse pressure. Values will be reported as the mean of three stable readings. This method has been shown to be reproducible in both healthy subjects and in patients with renal impairment [30,32]. The results for AIx will be corrected to a heart rate of 75 beats/minute (AIx_75_) [33]. All measurements will be made in triplicate and mean values used in analysis.

### Echocardiography

A comprehensive transthoracic echocardiogram (Vivid 7, GE Vingmed Ultrasound, Horten, Norway) will be performed with the subject in the left lateral decubitus position by a single experienced echocardiographer using second harmonic imaging and an M3S multi-frequency transducer. All parameters will be measured in triplicate and averaged as per the recommendations of the American Society of Echocardiography [34]. Analysis will be performed offline by a single blinded observer using an EchoPAC workstation (GE Vingmed Ultrasound, Horten, Norway). Ventricular dimensions, wall thickness, chamber volumes and stroke volume will be determined using standard methods [35]. Left ventricular mass will be determined by the 2-dimensional area-length formula and indexed to body surface area. Resting left ventricular diastolic function will be determined using standard techniques [36]. Peak systolic (s'), early diastolic (e') and late diastolic (a') mitral annular velocities will be measured at end expiration at the septal, lateral, inferior and anterior left ventricular walls with real time pulsed wave tissue Doppler [37].

Greyscale images for 2-dimensional left ventricular strain and strain rate imaging will be acquired in cineloop format in triplicate from the apical 4-, 2- and 3-chamber views and parasternal short axis views at basal, equatorial and apical levels at end expiration at frame rates >70/second for offline analysis using commercially available software (Speqle Tracking, GE Healthcare, United Kingdom). The endocardial border will be manually tracked at end-systole and the software will then generate a region of interest that will be placed over the myocardium. This will enable frame-to-frame tracking of ultrasonic speckles that change position according to surrounding tissue motion throughout the cardiac cycle. Peak systolic velocities, strain, strain rate, rotation and twist will be measured for each myocardial segment in triplicate and averaged.

### Dual Energy X-ray Absorptiometry Scanning

Dual energy x-ray absorptiometry scanning (Hologic QDR Series 4500 with Discovery Software version 11.02:03, Hologic Europe, Zaventem, Belgium) will be used to assess bone mineral density of the lumbar spine (L1-L4) and both proximal femurs (femoral neck, Ward's region, trochanteric region). Scans will be reported by an experienced bone densitometry clinical scientist blinded to clinical data.

### Run-in Phase

Following baseline studies all subjects will receive 1600 mg of sevelamer carbonate with meals for 4 weeks during an open-label run-in phase (Figure 1) to assess tolerability, efficacy, compliance and side effect profile. The development of new symptoms that may relate to treatment and compliance with medication will be assessed at 2-weekly intervals during run-in. Serum phosphate will also be measured at 2-weekly intervals. In the event that serum phosphate falls below 0.8 mmol/L, the dose of sevelamer will be halved to 800 mg with meals (Table 2) and levels rechecked to ensure serum phosphate is >0.8 mmol/L. If serum phosphate remains below this level after 6 weeks and/or a dose reduction subjects will be withdrawn from the study after a final set of measurements.

**Table 2:** Management of serum phosphate levels during open-label run-in phase

	Serum Phosphate (mmol/L)	Action
Week 2	<0.8	Reduce dose to 800 mg tds and continue to week 4
	>0.8	Continue to week 4

Week 4	<0.8	Reduce dose to 800 mg tds and continue to week 6 If already on 800 mg tds withdraw from study after week 4 visit
	>0.8	Randomise

Week 6	<0.8	Final measurements and withdraw from study
	>0.8	Randomise

### Randomisation and Treatment Phase

At the end of the open-label run-in phase subjects will attend for a second study visit and the following will be repeated: i) office brachial blood pressure and heart rate measurement in triplicate; ii) applanation tonometry to determine aortic pulse wave velocity; iii) applanation tonometry to derive central pressure waveforms from pulse wave analysis; iv) collection of serum and plasma for haematological and biochemical analysis; v) storage of serum and plasma at -80°C for future assay of biomarkers associated with cardiovascular function and calcification; vi) collection of a spot urine sample to determine albumin: creatinine ratio; and vii) 24-hour urine collection for determination of phosphate excretion.

Participants will then be randomised by computer assignment to continue 1600 mg (or half-dose) sevelamer with meals or receive an identical placebo for the remaining 36 weeks (Figure 1). During this blinded treatment phase subjects will undergo monitoring of renal function and serum calcium, phosphate and PTH every four weeks. Serum phosphate levels will be managed according to a pre-defined protocol during the treatment period (Table 3). All measurements performed at the initial main study visit (except for the plain lateral abdominal radiograph) will be repeated at a final visit 40 weeks after baseline studies, marking the end of subject participation in the study.

**Table 3:** Management of serum phosphate levels during treatment phase

	Serum Phosphate (mmol/L)	Action
At visit	<0.8	Recheck serum phosphate within 1 week
	>0.8	Continue routine 4-weekly checks

Within 1 week of visit	<0.8	Reduce treatment/placebo dose to 800 mg tds and recheck in 2 weeks
	>0.8	Continue routine 4-weekly checks

2 weeks after visit	<0.8	Final measurements and withdraw from study
	>0.8	Continue routine 4-weekly checks

### Subject Withdrawal

Withdrawal criteria are listed in Table 4. The average serum phosphate level of patients with stage 3 CKD enrolled in a previous study by the investigators was 1.2 ± 0.2 mmol/L [38]. It is therefore felt unlikely that significant numbers of participants will need to be withdrawn because of hypophosphataemia. The majority of patients with stage 3 CKD do not require phosphate-binding medication for overt hyperphosphataemia and the prevention of metabolic bone disease. Should any subjects in the placebo group develop serum phosphate >1.8 mmol/L, they will be withdrawn from the study and rescue therapy with appropriate phosphate binders will be administered.

**Table 4:** Withdrawal criteria

Withdrawal Criteria
Pregnancy
Hypophosphataemia (serum PO_4 _<0.8 mmol/L despite dose reduction of treatment/placebo)
Hyperphosphataemia (serum PO_4 _>1.8 mmol/L in subjects receiving placebo)
Occurrence of any serious adverse event, intercurrent illness or laboratory abnormality which, in the opinion of the investigators, warrants the subject's permanent withdrawal from the study
Poor compliance with study medication
Poor attendance at study visits
Subject inability to tolerate study medication due to side effect profile
Subject decision to withdraw
Deteriorating renal function
Subject lost to follow-up (loss of contact before final study visit)

### Endpoints

The primary end-point of the study will be a reduction in left ventricular mass after 40 weeks of treatment. Left ventricular mass is a powerful predictor of cardiovascular and all-cause mortality in the general population[39] and in CKD [40]. A therapeutic reduction in left ventricular mass has been shown to effectively reduce cardiovascular morbidity and mortality [41,42]. Secondary end points are: i) change in aortic compliance measured by CMR; ii) change in arterial stiffness as assessed using pulse wave velocity and pulse wave analysis; iii) change in arterial elastance measured by echocardiography; iv) change in left ventricular systolic and diastolic elastance measured by echocardiography; v) change in left ventricular function as assessed by tissue Doppler imaging, strain and strain rate analysis and measurement of torsion; and vi) change in bone density on DEXA scanning and/or magnetic resonance imaging.

### Planned Statistical Analysis

Comparisons will be performed between the groups at baseline and after the 36-week treatment phase. The normality of distribution of all continuous variables will be determined using a normality plot and the Kolmogorov-Smirnov test. Normally distributed variables will be analysed using unpaired t-tests, χ^2 ^or repeated measures analysis of variance with corrections made for multiple comparisons. Variables not normally distributed will be log transformed prior to analysis to achieve normal distribution or, if this is not achieved, analysed by Mann-Whitney U or Kruskal-Wallis tests. Multiple linear regression models will be derived for left ventricular mass and measures of vascular and cardiac stiffness using stepwise regression analysis. Analysis will be by intention-to-treat. A p-value of <0.05 will be considered statistically significant.

Sample size calculations were based on the primary endpoint of change in left ventricular mass after 40 weeks of treatment. A previous study by our group evaluating the cardiovascular effects of spironolactone in patients with early CKD revealed a 14 g drop in left ventricular mass from baseline following 40 weeks of therapy [38]. Using data from this previous study, 55 subjects in each arm will provide at least 80% power of detecting an 8 g difference between the change in left ventricular mass from baseline to 40 weeks using a two-tailed t-test at the 5% significance level given a standard deviation of 15 g in the change in left ventricular mass from baseline. Recruiting 60 subjects to each group will allow a 10% withdrawal or dropout rate.

### Monitoring and Safety Assessments

All adverse events, including serious adverse events (SAEs), will be recorded and followed up for the duration of the study or until resolution. Assessment of adverse events will be performed by the study investigators. All SAEs will be graded and reported to the sponsor. Any suspected unexpected serious adverse reactions will be reported to the sponsor, ethics committee and Medicines and Healthcare products Regulatory Agency. This study has been reviewed and approved by West Midlands Research Ethics Committee. Written informed consent will be obtained from all study participants.

## Summary

The demonstration of an important pathophysiological role for serum phosphate in the development of cardiovascular disease associated with CKD would be crucial in the understanding of this condition and have important implications for treatment. As left ventricular mass and arterial stiffness are prognostically significant markers in CKD, a positive effect would suggest that phosphate lowering in stage 3 CKD with the non-calcium-based phosphate binder sevelamer, in addition to conventional treatment for blood pressure and hypercholesterolaemia, is of prognostic value and would provide a rationale for a large clinical trial with cardiovascular morbidity and mortality as end-points.

## Abbreviations

Aix: augmentation index; AIx_75_: augmentation index corrected to a heart rate of 75 beats/minute; CKD: chronic kidney disease; CMR: cardiac magnetic resonance; DEXA: dual energy x-ray absorptiometry; ECG: electrocardiogram; ESKD: end stage kidney disease; GFR: glomerular filtration rate; PTH: parathyroid hormone; SAEs: serious adverse events

## Competing interests

The sponsor for this trial is University Hospitals Birmingham NHS Foundation Trust, Birmingham, UK. This investigator-led study is funded through an unrestricted educational grant from Genzyme Corporation, Cambridge, Massachusetts. Genzyme Corporation manufactures sevelamer carbonate (Renvela^®^), a non-calcium-based phosphate binder currently licensed in Europe for the treatment of hyperphosphataemia in dialysis patients and CKD patients not on dialysis with serum phosphate >1.78 mmol/L, which has been provided free of charge for the purposes of this study. In addition CJF has received lecture and advisory board fees from Genzyme. No employee from Genzyme or any medical writers have been involved in the preparation of this manuscript.

## Authors' contributions

CJF, JNT, RPS and CDC all contributed to the design of the study, the drafting of the protocol and this manuscript. CDC will acquire the majority of the data. All authors will be involved in statistical analysis and data interpretation. All authors have read and approved the final version of the manuscript.
